# Organizational complexity and demographic scale in primary states

**DOI:** 10.1098/rsos.171137

**Published:** 2018-05-02

**Authors:** David S. Sandeford

**Affiliations:** School of Human Evolution and Social Change, Arizona State University, Tempe, AZ 85281, USA

**Keywords:** social evolution, complex networks, comparative archaeology, state formation

## Abstract

The relationship between organizational complexity and demographic scale is an enduring research problem at the intersection of the natural and social sciences and has far reaching implications for the study of social evolution, particularly the emergence and collapse of complex social organizations such as chiefdoms, states and empires. Anthropological models of social evolution universally assume that population growth plays a critical role in the development of organizational complexity; however, the relationship between organizational complexity and demographic scale has not been formalized and cross-culturally validated. There is a rich yet unsystematized body of diachronic organizational and demographic data describing the evolution of organizational complexity in 10 archaeologically known cases of primary state formation. Using this dataset, this essay proposes and tests a complex network model that describes state societies as discrete, self-similar, hierarchical social networks. The model accurately describes how organizational complexity and population scale in all cases. The complex network architecture of state societies suggests that further advances in our understanding of modern social organization may be found by a deeper investigation of the role of human nature in the evolution of human societies.

## 1. Introduction

The relationship between organizational complexity and demographic scale is an enduring research problem at the intersection of the natural and social sciences and has far-reaching implications for the study of social evolution, particularly the emergence and collapse of hierarchical social organizations such as chiefdoms, states and empires. In the late eighteenth century, Adam Smith argued that organizational complexity developed in proportion to the integrative mechanism of the market which, in turn, developed ‘in proportion to the … populousness of’ a nation. ‘The nations that … appear to have been first civilized’ were characterized by greater organizational complexity relative to their ‘barbarous and uncivilized’ contemporaries due to their larger populations integrated by riverine markets [1, pp. 21–5]. Smith’s observation was taken as axiomatic by the evolutionary anthropologists of the late nineteenth and mid-twentieth centuries and their intellectual heirs in processual archaeology [2–10]. The theoretical eclecticism of contemporary models of social evolution tends to occlude the fact that these models uniformly assume that population dynamics underwrite—or are at least the best index of—the emergence and collapse of organizational complexity [11–25].

Despite this long-standing universal recognition, the formal relationship between organizational complexity and demographic scale remains at the level of empirical description rather than theoretical explanation [26]. Various upper and lower demographic limits have been proposed for human societies of differing levels of complexity; however, the proposed thresholds have little to no diagnostic, explanatory or predictive power [27]. At best, they are rough approximations of the order of magnitude of the population, usually expressed as a function of the levels of political hierarchy or social-evolutionary type (e.g. [15, table 8],[22, pp. 171–177, fig. 2], [25], [28], [29, pp. 294–297], [30, pp. 26–27], [31, table 14.1], [32, pp. 40–44, tables 2–4], [33]). The complex network model of organizational complexity and demographic scale outlined in this paper provides a theoretical framework that predicts and explains both the *qualitative* shift from egalitarian to hierarchical social organization and the *quantitative* relationship between organizational complexity and population, at least in all archaeologically known cases of primary state formation.

## 2. The complex network model

### 2.1. Assumptions of the complex network model

The complex network model of organizational complexity and demographic scale makes two fundamental assumptions about complex human social organization. These assumptions are extrapolated from analyses of large-scale ethnographic datasets that describe the dynamics of social networks in small-scale societies.

The first assumption is that the size of stable egalitarian social groups is limited. The cost of maintaining cooperation and coordination within and among social groups (‘scalar stress’) is an exponential function of population. As the the size of a social group increases, the capacity to maintain face-to-face, egalitarian means of group integration is quickly outstripped [34]. Research conducted under the rubric of the social brain hypothesis confirms that the sizes of egalitarian social networks in humans (*N*=5,15,50,150) are a function of and ultimately constrained by the capacity to process social information. It suggests that the demographic limit to non-hierarchically integrated forms of social organization is approximately 150 persons [35–37]. Higher-order groups (*N*>150) require hierarchical decision-making, coercion and other structural forms of political inequality or institutional means of group integration to maintain coherence and cooperation over time [36].

The second assumption is that complex societies can be described as discrete, self-similar, hierarchical social networks [38]—in other words, as complex networks. Statistical analyses of mobile foraging and hunter–gatherer groups suggest that human populations are distributed among a hierarchy of discrete social group sizes (*G*_*L*_) characterized by a self-similar branching or scaling ratio (*S*) [39–41]. The group-sizes *G*_*L*_ and the branching ratio *S* are conceptually identical to the Horton orders *g*^(*ω*)^ and the Horton–Strahler branching ratio *B* used by Hamilton *et al.* [40]. The empirical values for these discrete group sizes are approximated by the series *G*_*L*_=1,5,15,50,150,500 for *L*=0,…,5, and correspond to the organizational units (*G*_*L*_) and levels of integration (*L*) observed in the ethnographic and historical record for individuals, nuclear families, hamlets, small villages, large villages, and ‘supravillages’, and, at higher levels of integration, urban central places of increasing size [15].

**Figure 1.**
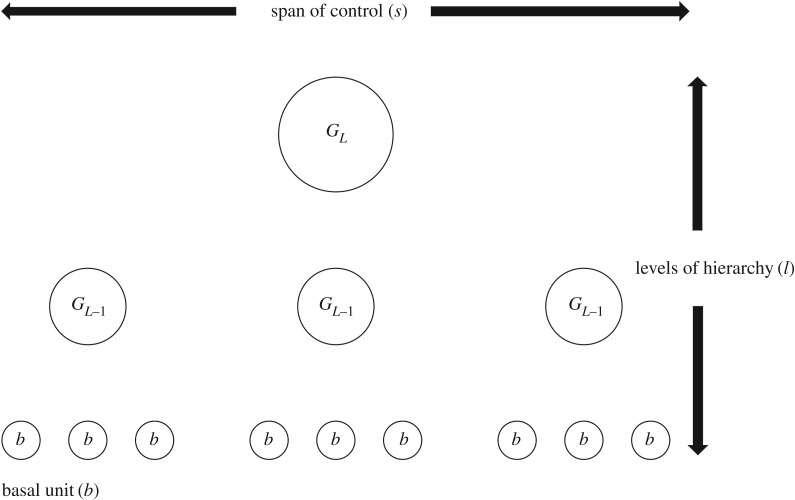
Parameters of organizational complexity. In this illustration, *s*=3, *l*=3 and *b*≥1.

There is a conceptual concordance between the societal parameters used in the ethnographic literature discussed above and in the archaeological literature on state formation. Both describe social organization in terms of three parameters: the levels of group integration (*L*) and levels of settlement hierarchy (*l*), both of which are metrics of organizational or social-structural *depth*; the branching ratio (*S*) and span of control (*s*), both of which describe organizational or social-structural *width*; and the basal unit which measures the number of persons per group at the *B*th level of group integration (*G*_*B*_) or the *l*th level of the settlement hierarchy (*b*) (figure 1). The basal unit of analysis (*b*) in the ethnographic studies cited above is implicitly taken to be the individual (*G*_0_); however, a generalized redefinition of the basal unit, *G*_*B*_=*G*_0_*S*^*B*^ for *B*<*L*, is possible given the self-similar scaling of social group sizes [42].

### 2.2. Methods for calculating societal parameters from archaeological data

Following the theoretical assumptions of systems theory [43] and economic geography [44], archaeologists generally partition the sites of a sociopolitical region into a hierarchy of discrete levels based on the relative sizes and frequencies of sites within the region and the overall geometry of its settlement pattern. Although no universally applicable diagnostic criteria or unambiguous mathematical methods have been developed to determine the number and exact boundaries of these levels, archaeologists generally partition a region based on the modes evident in the site-size distribution [45] or its logarithmic transformation [46], both of which are proxies for the distribution of discrete groups sizes (*G*_*L*_) in a region. Unless an author has deviated from this standard practice or newer evidence compels revision, the levels of settlement hierarchy (*l*) and their boundaries are taken directly from the sources cited below.

**Figure 2.**
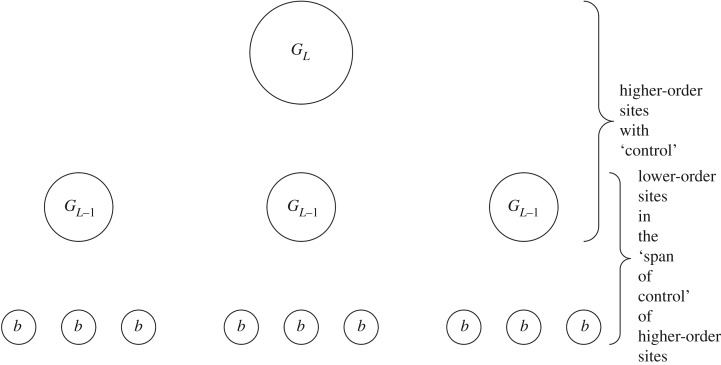
Span of control. In this illustration, *s*=3, *l*=3 and *b*≥1. *N*(*G*_*B*_)= 9, *N*(*G*_*B*+1_)=3, *N*(*G*_*B*+2_)=1. *s*=(*N*(*G*_*B*+0_)+*N*(*G*_*B*+1_))/(*N*(*G*_*B*+1_)+*N*(*G*_*B*+2_))= (9+3)/(3+1)=3.

The span of control (*s*) is the ratio of lower-order to higher-order groups in a given society [34,47]. It is equivalent to the branching ratio (*S*) discussed above [39,40]. There is no standard method for calculating an average societal span of control; however, equation (2.1) is equivalent to iterating over all sites in a region that have ‘control’, counting how many sites are in their direct ‘spans of control’, and averaging this number (figure 2):

2.1 $$s=\frac{\sum_{i=0}^{l-2}N(G_{B+i})}{\sum_{i=1}^{l-1}N(G_{B+i})},$$

where *N*(*G*_*B*+*i*_) is the number of groups at settlement level *i*+1.

The size of the basal unit (*b*) is the number of persons in the organizational unit that occupies the lowest level in the settlement hierarchy [34,48]. It is calculated by multiplying the minimal regional population density estimate [49] ($\rho_{\min})$ by the average size of the lowest-level sites (${\bar{s}}_{b}$):

2.2 $$b=\rho_{\min}{\bar{s}}_{b}.$$

The demographic growth rate (*r*) is the annualized percentage change in a region’s population. It is calculated as

2.3 $$r=\frac{\ln(A_{2}/A_{1})}{t_{2}-t_{1}},$$

where *A*_2_ and *A*_1_ are the total settlement areas at times *t*_2_ and *t*_1_, which mark the end and beginning of the period in question. Population estimates at *t*_2_ and *t*_1_ can be substituted for the total settlement area.

The archaeologically estimated population range was calculated by multiplying the total settlement area *A* by the minimal and maximal regional population densities, $\rho_{\min}$ and $\rho_{\max}$:

2.4 $$[N_{1},N_{2}]=[A\rho_{\min},A\rho_{\max}].$$

The data and calculations used for the case studies below are catalogued in the spreadsheets submitted as electronic supplementary material, S1. Any ad hoc calculations are detailed in the footnotes.

### 2.3. Predictions of the complex network model

The complex network model makes three general predictions. One, in accord with the social brain hypothesis, it predicts basal units of approximately 5, 15, 50 or 150 persons and autonomous villages of no larger than 150 persons:

2.5 b∈{5,15,50,150}.

Once an autonomous village or group of villages reaches a critical level of ‘scalar stress’, it will either fission or elaborate hierarchical, institutional means of group integration [50]. The development of organizational hierarchy is directly observable in the development of discrete ‘levels’, ‘orders’ or ‘tiers’ of sites of increasing size and decreasing frequency in a region’s settlement pattern.

The social networks of complex societies retain the modular and self-similar scaling properties that characterize the social networks of small-scale societies. However, because sedentary populations are not characterized by the fission–fusion dynamics of mobile foraging and hunter–gatherer groups [51], the total population of a region is not equal to *G*_*L*_ but to the sum of *G*_*L*_ for all levels:

2.6 $$N=\sum_{i=0}^{l}bs^{i}.$$

Because the size of the nuclear family can be expressed as a function of the net reproductive rate (*G*_2_=2(*R*+1)), the branching ratio or span of control of a complex society can be predicted given the demographic growth rate, *r*, where R=exp⁡[rτ] and *τ*, the average generation span, is assumed to be 20. Given an estimated range of the population independent of equation (2.6), the third general prediction of the model is expressed as

2.7 $$s=\sqrt[{L}]{N(G_{2})},$$

where *L* is the level of group integration above the nuclear family (*L*=*l*+*B*−2) and *N*(*G*_2_) is the number of nuclear families in the population (*N*(*G*_2_)=*N*/2(*R*+1)) (derived from [40, eqn 5.2]).

## 3. Case studies

The validity of the complex network model is assessed by examining organizational complexity and demographic scale in all archaeologically known primary states. Anthropologists have minimally defined states as socially stratified and bureaucratically governed societies with at least four levels of settlement hierarchy [52]. Primary states are those states developed by strictly autochthonous processes and interaction between organizationally similar non-state societies [53,54]. The comparative analysis below documents the dynamics of the demographic and organizational parameters of the societies in question, following the neoevolutionary arc from autonomous villages, to simple and complex chiefdoms, to states [55,56]. It confirms the fundamental role that demographic growth plays in the process of social evolution and suggests that the rate of demographic growth (*r*) is related to the organizational structure of complex societies to a degree not previously recognized (equation (2.7)).

### 3.1. Susa, southwestern Iran, *ca* 4000–3000 BCE

At the turn of the fourth millennium bce, the Susiana Plain of southwestern Iran was populated by 18 ‘largely autonomous’ villages of no more than 150 people. There is no evidence from this period of political authority beyond the level of local elders [57, pp. 43, 100]. Over the next 500 years, the population tripled, increasing at a rate of *r*=0.001, and the region sequentially developed two and three levels of settlement hierarchy. By 3500 bce, there is clear evidence of social stratification, bureaucratic governance and the development of a four-tiered population distribution centred around Susa [58, pp. 101–141].

Johnson [58, table 18 and fig. 10] partitions the settlements into 37 large villages, 13 supravillages, two small centers and Susa yielding a span of control of 3.25. The basal unit in southwest Iran was the large village of approximately 150 persons.^1^ The archaeologically estimated population ranges from 12 783 to 25 556 persons [45,58]. The complex network model predicts a population of 24 106 persons and a span of control of 3.16 to 3.49.

### 3.2. Uruk, southern Iraq, *ca* 4000–3000 BCE

Agricultural villages emerged on the southern Mesopotamian alluvial plain during the sixth millennium bce. For the next 1000 years, the region was characterized by an ‘extremely low population and settlement density’ of no more than 15 autonomous villages ‘widely and fairly evenly dispersed’ across the plain. This pattern prevailed until roughly the end of the fifth millennium bce when a ‘striking increase in population’ following the collapse of the Susa state and mass emigration from the Susiana plain [24, pp. 453–59] kick-started what Adams describes as ‘the processes of precocious growth that (led to) the development in southern Mesopotamia of the world’s earliest civilization’ [59, pp. 55–60]. As the population of the southern alluvium grew by a factor of roughly eight over the next millennium (*r*=0.005), the region sequentially developed two, three and four levels of settlement hierarchy, marked social stratification, and institutions of bureaucratic governance [60, pp. 65–127].

**Figure 3.**
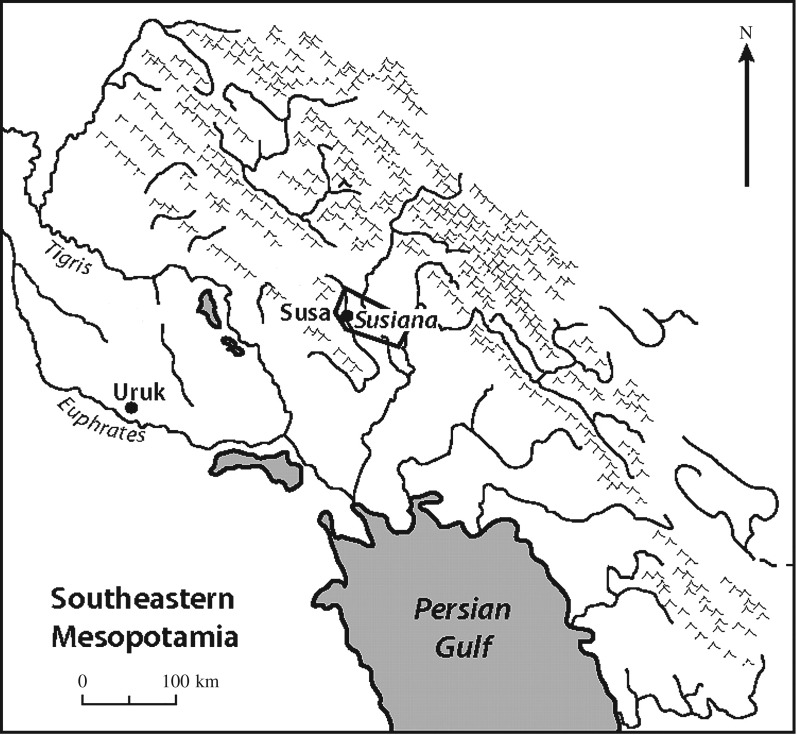
Uruk and Susa, southern Mesopotamia *ca* 4000–3000 BCE (adapted from Spencer [23, fig. 7]).

The Uruk population was distributed among 77 large villages, 18 supravillages, five small centers, and the city of Uruk yielding a span of control of 4.17.^2^ The average size of the large village in southern Mesopotamia was approximately 150 persons.^1^ The archaeologically estimated population of Uruk is 54 070 to 108 140 persons. The complex network model predicts a population of 59 441 persons and a span of control of 3.85 to 4.26 (figure 3).

### 3.3. Hierakonpolis, upper Egypt, *ca* 3500–3100 BCE

The earliest complex societies in Egypt also emerged in the fourth millennium bce. This period of ‘rapid social and political evolution’ witnessed the ‘transition from autonomous villages to an early state’ [61, pp. 267, 282]. These autonomous villages were on average 150 persons in size [62, p. 155]. Climatic change and population nucleation and growth precipitated the rise of the Hierakonpolis state (*r*=0.001) [63, table 4]. Rainfall became increasingly rare during the fourth millennium bce leading to the abandonment of settlements on the desert margins and the nucleation of population on the banks of the Nile [63]. Hoffman [64, pp. 309–11] suggests that these demographic dynamics were acute in upper Egypt, particularly in the region surrounding Hierakonpolis, providing the conditions under which bureaucratic governance would become necessary and social stratification possible.

**Figure 4.**
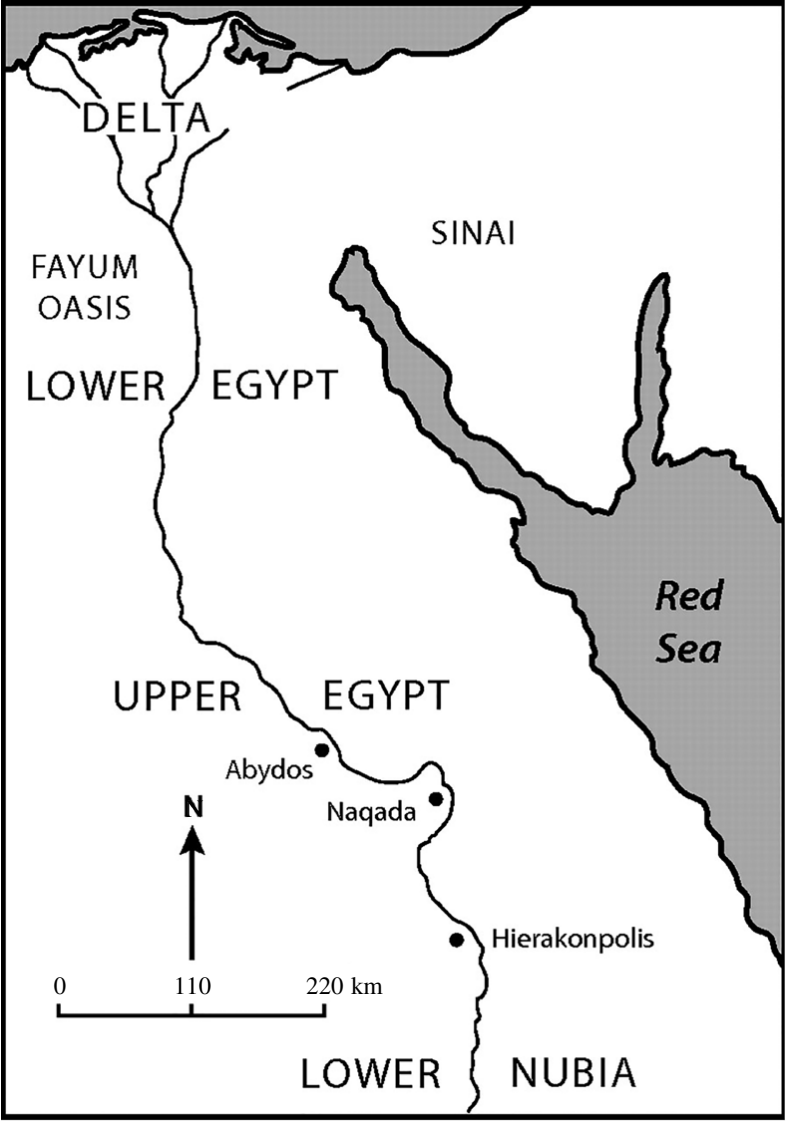
Upper Egypt, *ca* 3500–3100 BCE (adapted from Spencer [23, fig. 6]).

The population of upper Egypt was distributed in a four-tiered hierarchy [65, table VI.4]: 17 large villages, three supravillages, three towns and Hierakonpolis [66, tables 23, pp.10–11] yielding a span of control of 3.29. Wenke [67, pp. 202–229] argues that the emergence of the Hierakonpolis state followed the bellicose expansion of the chiefdom of Hierakonpolis which conquered and incorporated the chiefdoms of Nagada and Abydos to its north. Assuming these political boundaries are approximately correct (figure 4), the archaeologically estimated population of the Hierakonpolis state is 12 500 to 30 000 persons.^3^ The complex network model predicts a population of 25 183 and a span of control of 3.15 to 3.57.

### 3.4. Harrapa, eastern Pakistan, *ca* 2600–2000 BCE

**Figure 5.**
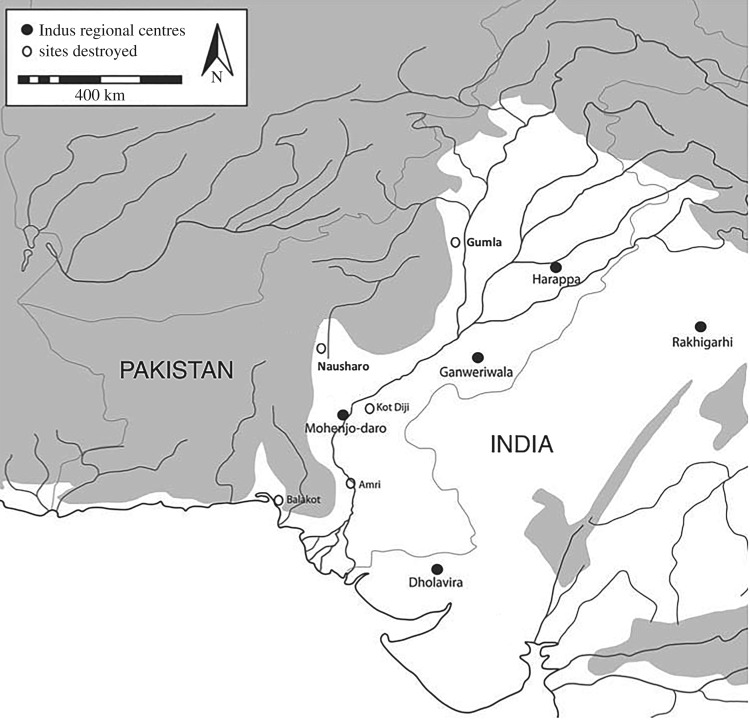
The Indus Valley *ca* 2600–1900 BCE (adapted from Coningham & Young [72, fig. 6.2]).

Prior to the turn of the third millennium bce the Indus Valley of eastern Pakistan was sparsely populated by ‘egalitarian social and settlement systems’ of autonomous agricultural villages [68]. Growing at an annualized rate of *r*=0.002, the population of the Indus Valley roughly tripled over the course of the third millennium and regional settlement patterns developed two- and three-level population distributions ([69, table 8.1],[70, pp. 79–144]). The emergence of state-level organization centred at Mohenjo-daro followed the razing of several competing chiefly centres [69] and widespread violence as far northeast as Harrapa [71] (figure 5). By 2600 bce, there is clear evidence for social stratification, bureaucratic governance, and a four- or five-level settlement hierarchy centred at Mohenjo-daro [73,74].

Mughal [75, p. 6] suggests applying the organizational scheme used by Adams [59, table 14] in southern Mesopotamia to the the Harrapan settlement pattern. This cross-cultural scheme partitions the Harrapan settlement distribution into five levels: 829 large villages, 113 supravillages, 68 small centres, 11 regional centres and Mohenjo-daro, yielding a span of control of 5.29 [76], appendix A.^4^ The basal unit of the Harrapan state is the large village of approximately 150 persons. The archaeologically estimated population of the Harrapan state ranges from 384 500 to 769 000 persons ([69, table 1],[70, pp. 107–110]). The complex network model predicts a population of 766 318 persons and a span of control of 5.14 to 5.78.

### 3.5. Erlitou, central China, *ca* 1900–1500 BCE

**Figure 6.**
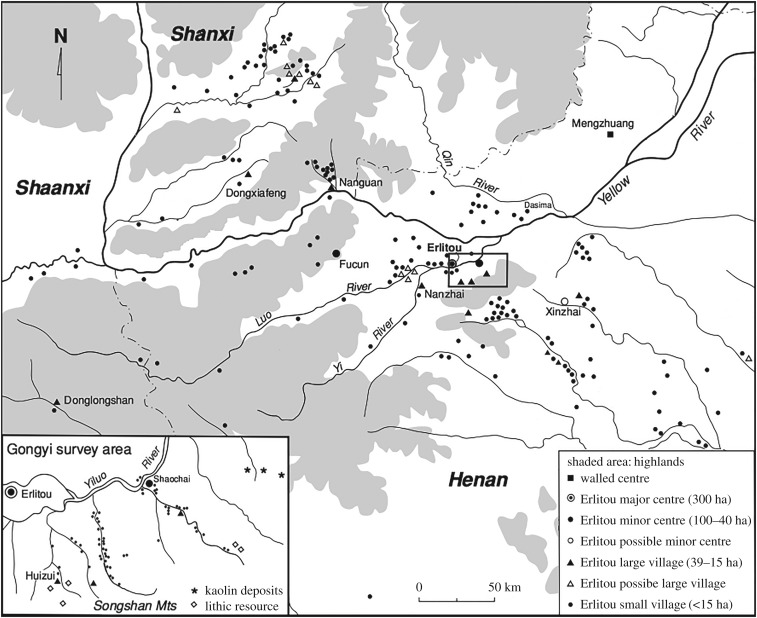
Erlitou sites on the central plains of China, *ca* 1900–1500 BCE (adapted from Liu [77, fig. 8.1]).

The Yiluo River Basin is a semi-circumscribed and agriculturally rich alluvial basin in the central plains of northern China spread between the modern provinces of Shanxi and Henan (figure 6). Over the course of the third millennium bce, this region exhibited continuous population growth (*r*=0.001), developing from a set of nine ‘relatively egalitarian’ villages of no more than 150 persons [78, pp. 84–5], to a two- and three-tiered chiefdom, to a socially stratified and bureaucratically governed state characterized by a four-level settlement hierarchy centred on Erlitou [77, pp. 159–191, 223–238].

The Erlitou population was distributed over four settlement levels: 148 large villages, 43 supravillages, 10 small centres and Erlitou itself yielding a span of control of 3.74 [77, fig. 8.2]. The basal unit of the region was the large village of approximately 150 persons.^5^ The archaeologically estimated population ranges from 27 841 [80, table 9] to 82 085 persons [79, table 9-4]. The complex network model predicts a population of 40 032 and a span of control of 3.53 to 4.12.

### 3.6. Monte Albán, southern Mexico, *ca* 300 BCE–200 CE

At the turn of the second millennium bce, the highlands of southern Mexico were sparely populated by autonomous small villages of approximately 50 persons [81] and there is no evidence from the Valley of Oaxaca indicating that any village was larger than 150 persons [82, pp. 25, 238]. The population of the Oaxaca valley tripled prior to the initial phase of state formation *ca* 300 to 100 bce and in the two centuries that followed the region’s population continued to grow at a rate of *r*=0.003 [83, table A.III.2]. This demographic profile corresponds to the emergence of the socially stratified, bureaucratically governed, four-level Monte Albán state between 300 and 100 bce and its rapid territorial expansion between 100 bce and 200 ce [46,82].

**Figure 7.**
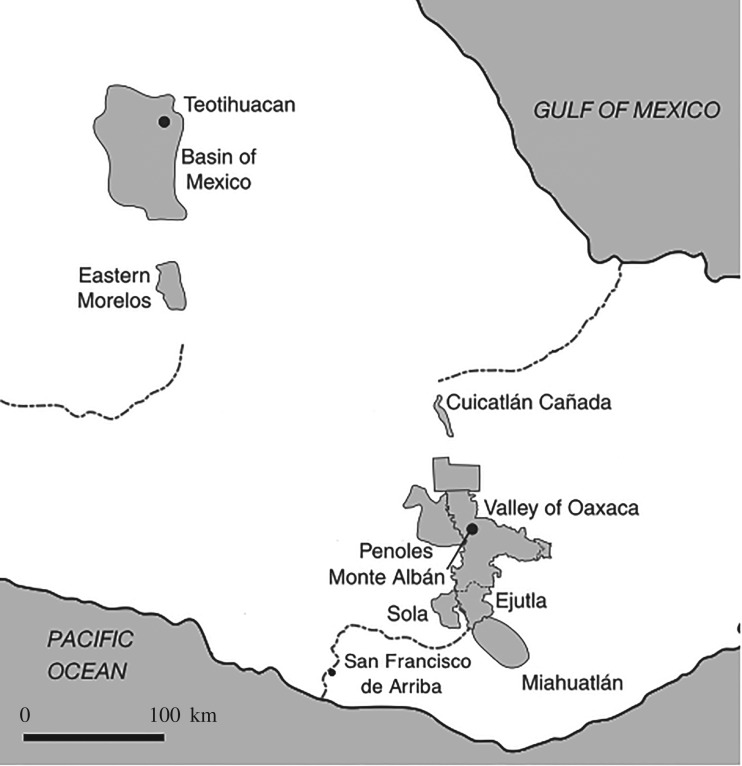
Monte Albán and Teotihuacan, central Mexican highlands, *ca* 100 BCE–200 CE (adapted from Feinman & Nicholas [84, fig. 3.1]).

At its territorial zenith, the Monte Albán state encompassed of the valleys of Oaxaca, Sola, Ejutla, Miahuatlán and Río San Francisco, the Peñoles and Guirún regions flanking the Oaxaca Valley, and the Cañada Cuicatlán to its north (see figure 7; [83], [85, table C.2], [84, table 6.3], [86, table 2-1], [87, pp. 255–256], [88, table 6.1], [89], [90, table 24]). The hamlet of approximately 15 persons was the basal unit of the Monte Albán state [84]. To integrate the regional demographic data outlined above and to account for the basal unit of 15 persons, the settlement levels given by Kowalewski *et al.* [83, fig. 7.9] have to be updated: levels 3 and 4 are merged into level 3 in order to maintain 5 total levels; levels 4 and 5 are partitioned at *N*=50 to account for the size of the basal unit. This yields 485 hamlets, 75 small villages, 45 large villages, six supravillages and Monte Albán and a span of control of 4.81. The archaeologically estimated population of the polity at this time is 32 530 to 67 860 persons. The complex network model predicts a population of 48 802 persons and a span of control of 4.46 to 5.04.

### 3.7. Teotihuacan, central Mexico, *ca* 100–1 BCE

Near the middle of the second millennium bce the highland and semiarid Basin of Mexico was colonized by sedentary farmers from the neighbouring Morelos region (figure 7). The initial settlements consisted of small autonomous villages and hamlets of less than 150 persons [91,92]. Over the course of the next 14 centuries, this population of a few hundred people grew several orders of magnitude at an annualized rate of *r*=0.003 [93, p. 183]. The dynamics of the Basin’s settlement pattern—the sequential development of two, three and four levels of settlements centred about segregated subregional centres—suggests the concurrent development of competing chiefly polities followed by the emergence of regional state-level organization *ca* 100–1 bce ([93, pp. 91–108], [13, pp. 111–28]). Cowgill [94, p. 55] argues that in addition to four levels of settlement hierarchy, there is strong evidence for institutionalized stratification and offices of bureaucratic governance in the Basin of Mexico during the last century bce, all of which suggest the presence of state-level organization at this time.

The Teotihuacan population was distributed among four levels of settlements [93, p. 102]: 200 hamlets and small vilages, 23 large villages, 10 regional centres, and Teotihuacan yielding a span of control of 6.85 [91,92].^6^ These hamlets and small villages were approximately 50 persons in size [91,92]. The population of the basin is estimated to range from 57 358 [91,92] to 175 000 [13, p. 112] persons. The complex network model predicts a population of 128 895 and a span of control of 5.12 to 6.03.

### 3.8. Virú, northern Peru, *ca* 200 bce–200 ce

By the latter half of the second millennium bce, the northern lowland coasts and the southern sierra highlands of Peru were populated by ‘innumerable autonomous collectives sustained by independent farming systems’. The onset of protracted drought around 900 bce halted demographic growth and the region’s level of organizational complexity remained at the level of ‘self-governing collectives’ of roughly 150 persons for another 1000 years [95, pp. 134–5, 174–6]. Quickly after demographic growth resumed *ca* 200 bce (*r*=0.003), the autonomous communities of the Virú Valley were rapidly transformed into the first state society in South America, evidenced by the emergence of four levels in Virú’s settlement pattern, social stratification and bureaucratic governance [96–98].

The population of Virú was distributed among four levels: 37 small villages, seven large villages, volume supravillages and the Gallinazo Group [99], appendices A–C yielding a span of control of 4.60. The basal unit of Virú was the small village of approximately 50 persons. The archaeologically estimated population of the Virú state ranges from 11 863 to 42 708 persons [99, table 5.1]. The complex network model predicts a population of 36 759 and a span of control of 3.77 to 4.67.

### 3.9. Tiwanaku, northern Bolivia, *ca* 300–600 CE

**Figure 8.**
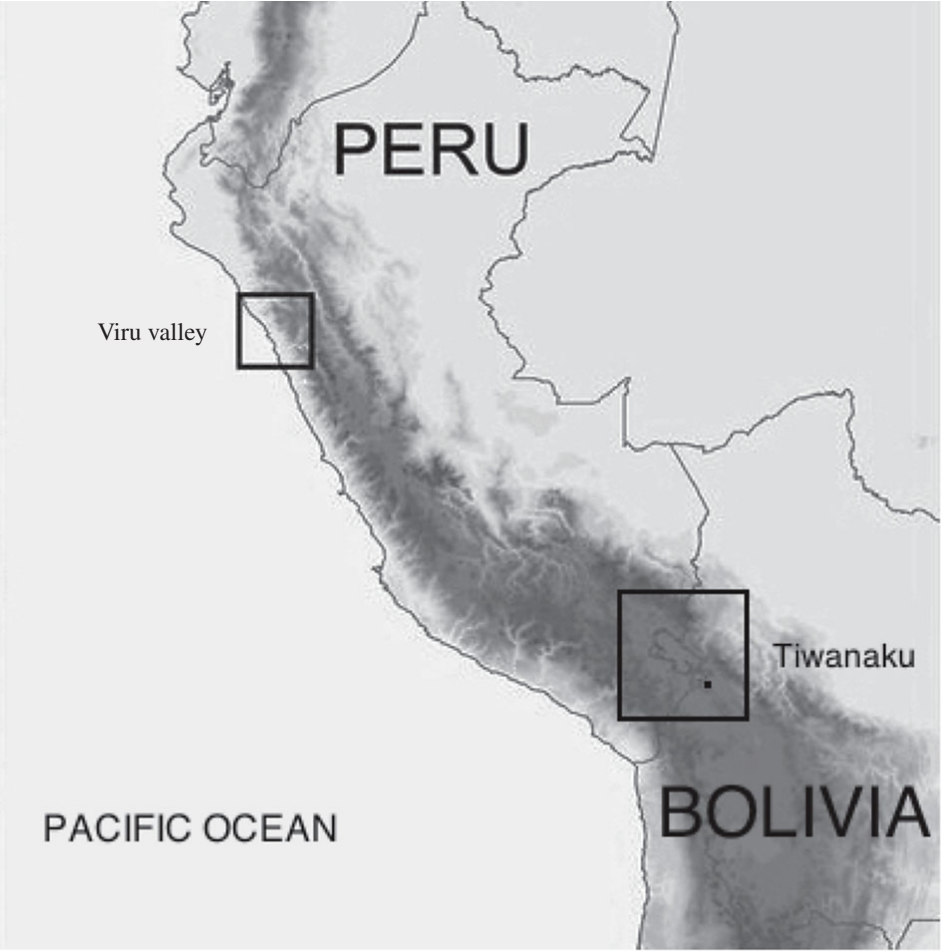
Peruvian lowlands and Bolivian altiplano, South America (adapted from Millaire *et al*. [98, fig. 1]).

In the second millennium bce the Bolivian altiplano surrounding Lake Titicaca (figure 8) was populated by a handful of ‘hamlets and small villages’ for which there is ‘no evidence of political ranking’. These autonomous villages were no larger than ‘a few dozen households’, that is they were smaller than 150 persons on average [100, pp. 100–8].^7^ At the turn of the first millennium in the Taraco Peninsula, there is strong evidence that population growth set into motion the fissioning of several large autonomous villages in which the population had surpassed 150. This process continued until the geographical landscape was completely saturated at a maximal level of ‘scalar stress’ [50]. As secular increases in population continued, the Titicaca Basin sequentially developed two, three and four levels in its population distribution [100–102]. Demographic growth accelerated prior to the emergence of the socially stratified and bureaucratically governed Tiwanaku state [103], the population nearly tripling from 300 to 500 ce (*r*=0.009).

The territory of the Tiwanaku state *ca* 500–600 ce consisted of the Taraco Peninsula and the Katari, Lower and Middle Tiwanaku valleys [104]. Settlement pattern and demographic data were compiled, respectively, from [105], appendix A, [106, appendix 6.1], [107, appendix 2] and [108, appendix A]. McAndrews *et al.* [102, fig. 10] partition these settlements into four levels: small villages, large villages, supravillages and Tiwanaku. When the Katari Valley survey data are added, a fifth level (small centres) must be added. This yields a distribution of 347 small villages, 69 large villages, 25 supravillages, nine small centers and Tiwanaku and a span of control of 4.32.

No regional population density or total population estimates are available for the nascent Tiwanaku state. However, using an estimated population of 60 000 for the city of Tiwanaku [109, p. 26] and following the population distribution for the city of Tiwanaku and its hinterland suggested by Bandy [104, p. 82], the estimated population of the Tiwanaku state is approximately 90 000 to 130 000 persons. This yields a regional population density estimate of 155 persons per hectare and a basal unit of approximately 50 persons. The complex network model predicts a population of 98 613 and a span of control of 4.13 to 4.37.

### 3.10. Hawai‘i, *ca* 800–1800 ce

**Figure 9.**
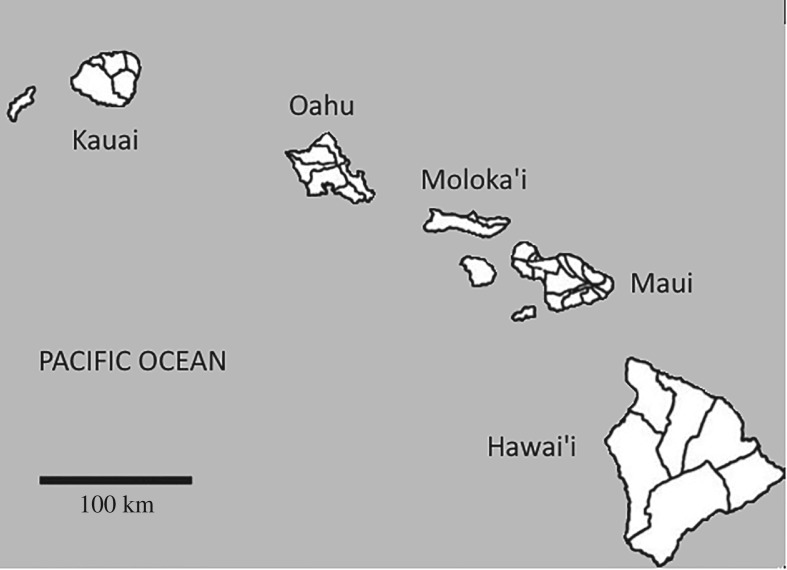
Hawaiian islands with districts, *ca* 1800 CE (adapted from Hommon [111, fig. 1.1]).

The emergence of social complexity in the Hawaiian islands is an important case study for several reasons. The Hawaiian islands are culturally and geographically separated from the complex societies of Eurasia and the Americas by several millenia and thousands of transoceanic miles. Polynesia was the last region on Earth to be colonized by human beings and the Hawaiian archipelago was the last of Polynesia to be settled. The first Hawaiian society was founded by no more than 100 people *ca* 800 ce [110, p. 12]. In the scope of less than 1000 years, this tiny neolithic colony evolved into an large state society populated by hundreds of thousands of people (*r*=0.013). Because this endogenous process culminated as contact with literate societies was made, the Hawaiian case is the only instance of primary state formation that we have historical records for. Our knowledge of the administrative and territorial organization of the islands comes directly from primary ethnohistorical sources. Similarly, the demographic estimates for the Hawaiian state at contact come from modern ethnographic and census data. Thus, the Hawaiian case is in several ways the exception that proves the rules (figure 9).

The population of the Hawaiian state was organized in six levels of territorial and administrative hierarchy [111,112]: 1211 local communities, 360 supralocal communities, 107 subdistricts, 36 districts, eight island-wide superdistricts, and the royal court itself yielding a span of control of 3.36 [113, fig. 1 and table 1]. The most recent estimate of the size of the local community (the *ahupua‘a* or large *‘ili ‘aina*) comes from Cordy [114, tables 7.1–7.2]. If the two largest *ahupua‘a*—‘where high chiefs and rulers did reside’—are removed from the dataset, the average size of the local community is 150 persons. The estimated population of the Hawaiian state ranges from 250 000 [115, p. 323] to 525 000 persons [111, pp. 12, 62]. The complex network model predicts a population of 302 166 and a span of control of 3.36 to 3.65.

## 4. Summary and discussion

The organizational and demographic data from the primary states discussed above and the predictions of the complex network model are summarized in table 1. The complex network model accurately describes the relationship between organizational complexity and demographic scale in all cases. The model also accurately describes the relationship between the span of control and the demographic growth rate in nine of the 10 cases. The discrepancy between the model’s prediction in the Basin of Mexico and the empirically observed span of control is likely due to the presence of a large number of seasonal occupation sites surrounding Teotihuacan [93, p. 108]. If half or more of these temporary sites are removed from the Teotihuacan dataset, the model predicts the span of control in all cases.

**Table 1.** Summary of archaeologically known organizational and demographic data and the predictions of the complex network model.

state	*s*	*l*	*b*	*N* (estimated)	*r*	*s*_*CNM*_	*N*_*CNM*_
Susa	3.25	4	150	12 783–25 566	0.001	3.16–3.49	24 106
Uruk	4.17	4	150	54 070–108 140	0.005	3.83–4.23	59 441
Hierakonpolis	3.28	4	150	12 500–30 000	0.001	3.15–3.57	25 065
Harappa	5.29	5	150	384 500–769 000	0.002	5.14–5.68	766 318
Erlitou	3.74	4	150	27 841–82 085	0.001	3.53–4.12	40 032
Monte Albán	4.81	5	15	32 530–67 860	0.003	4.45–5.04	48 802
Teotihuacan	6.91	4	50	57 385–175 000	0.003	4.82–5.81	133,404
Virú	4.60	4	50	11 863–42 702	0.003	3.76–4.66	28 592
Tiwanaku	4.32	5	50	90 909–133 333	0.009	4.13–4.36	98 613
Hawai‘i	3.42	6	150	250 000–525 000	0.013	3.36–3.65	307 231

This paper provides a theoretical framework that predicts and explains both the qualitative shift from egalitarian to hierarchical social organization and the quantitative relationship between organizational complexity and population in the context of demographic growth. This framework treats demographic growth as strictly exogenous to the social system but it is clear that any satisfactorily comprehensive, comparative, quantitative and dynamic model of human social evolution must endogenize and explain the variation in growth rates in human societies. As was stressed above, the critical qualitative difference between egalitarian and hierarchically organized societies is sedentism. Comparative analyses of the origins of sedentism and the ‘neolithic demographic transition’ suggest that the energetic windfall provided by agricultural subsistence may explain both the transition from foraging to farming and the subsequent rates of demographic growth in sedentary societies [116,117]. If this reasoning is correct, then it follows that future models of social evolution must systematically incorporate and account for the general energetic properties of human societies and agricultural systems. A comparative energetic analysis along these lines may, for example, yield insights into the variability in the tempo of social evolution in the Pacific, Old and New Worlds. In the absence of migration, New World societies based on maize cultivation tended to grow roughly three times more quickly than Old World societies; Tiwanaku and Hawai‘i—both based on tuber cultivation—grew nearly 10 times as quickly. An energetic analysis may also help explain why New World state societies are uniformly characterized by smaller basal units.

The discrete, self-similar, hierarchical nature of the social network architecture of human societies explains the universality of distinct levels of urban settlement hierarchy and the observed branching patterns between higher- and lower-order sites in terms of the interaction between the scope and limits of our cognitive endowment, demographic growth and institutional development. The complex network model may help resolve the problem of the origins of urban hierarchies—what economic geographer Paul Krugman famously called the ‘striking empirical regularity with no good theory to account for it’ [118]. This suggests the need to further develop the mathematical methods used to detect complex social networks [39] and apply these to archaeological, historical, and contemporary urban settlement patterns. Above all, it strongly suggests that further advances in our understanding of modern social organization may be found by a deeper investigation of the role of human nature in the evolution of human societies.

## Supplementary Material

Settlement pattern data for primary states

## Footnotes

1 There is no consensus value for a regional $\rho_{\min}$ and the boundaries of level 4 of the settlement hierarchy are disputed both within and between subregions, giving alternative values for ${\bar{s}}^{l}$. At a minimum, $b=\rho_{\min}{\bar{s}}^{l}=100\;{\text{p⁡ha}}^{-1}\times 1.22\;\mathrm{ha}\approx 123$ persons; at a maximum, $b=\rho_{\min}{\bar{s}}^{l}=125\;{p\;\mathrm{ha}}^{-1}\times 1.39\;\mathrm{ha}\approx 175$ persons. These values give an average *b* of approximately 150 persons.

2 The Uruk settlement pattern data were compiled from Adams [59, table 7] and Algaze [20, appendix 2]. Algaze updated the size of Uruk from 100 to 250 ha but excluded sites less than 0.1 ha from Adams’s original dataset. Algaze also sets the boundary between levels 3 and 4 at 5 ha, where Adams had set it at 4 ha. To reconcile these discrepancies, I have increased the size of Uruk, kept the smaller sites in the dataset, and split the six sites between 4 and 5 ha evenly between levels 3 and 4.

3 For Butzer [63, tables 2–3]: estimates assume nomes 3–8 are cultivated for a total of 411.8 km^2^ of cultivated land and a population density of 30 to 70 persons per cultivated square kilometre of land, yielding 12 354 to 30 885 persons. For Hoffman [65, p. 141]: this figure assumes five persons per house, 0.00549 houses per square metre of settlement, and 453 580 km^2^ of settlement, yielding 12 451 persons. For Hassan [62, pp. 161–162]: this figure assumes six nomes and 4880 persons per nome, yielding 29 280 persons.

4 The settlement pattern data from Possehl [76, Appendix A] are incomplete. Of the 1022 known Mature Harrapan sites [69, table 8.1], only 513 are of known dimensions. This limitation is overcome by recent archaeological work that confirms that these unknown sites are less than 1 ha in size [72, pp. 200–202]. I have taken the average site size for all sites *s*<1 ha and substituted this value for the unknown site sizes.

5 This figure is based on an estimated village population density of 41–62 persons per hectare [79, p. 183] and 3 ha per large village. This estimated size of the large village is based on the modal distribution of the sites, not the demarcations given by Liu [79, fig. 8.2].

6 The regional centre Cuicuilco with a population of 20 000 persons was included in the compilation of sites for this period.

7 ‘A few dozen households’ is estimated to range from 24 households at four persons per household (96 persons) to 48 households at five persons per household (190 persons), or 143 persons per village on average.

## Data accessibility

The settlement pattern data for all states discussed above (with the exception of Hawaii) are available as electronic supplementary material, S1 (primary_states.xls).
